# Multiple pregnancy in SARS-CoV-2 outbreak: the prenatal care challenge

**DOI:** 10.31744/einstein_journal/2020CE5990

**Published:** 2020-09-16

**Authors:** Eduardo Felix Martins Santana, Julio Elito

**Affiliations:** 1 Faculdade Israelita de Ciências da Saúde Albert Einstein Hospital Israelita Albert Einstein São Paulo SP Brazil Faculdade Israelita de Ciências da Saúde Albert Einstein, Hospital Israelita Albert Einstein, São Paulo, SP, Brazil.; 2 Hospital Israelita Albert Einstein São Paulo SP Brazil Hospital Israelita Albert Einstein, São Paulo, SP, Brazil.; 3 Escola Paulista de Medicina Universidade Federal de São Paulo São Paulo SP Brazil Escola Paulista de Medicina, Universidade Federal de São Paulo, São Paulo, SP, Brazil.

Dear Editor,

In December 2019, the human history changed when a new virus was detected in the city of Wuhan, China. Millions of individuals were rapidly infected all over the world and the new coronavirus, initially called coronavirus disease 2019 (COVID-19), made a huge impact by causing severe acute respiratory syndrome; hence, it was later called severe acute respiratory syndrome coronavirus 2 (SARS-CoV-2). With a rapid transmission, in March 2020, the World Health Organization (WHO) declared the infection a pandemic. People on all continents have been affected and the number of deaths has been increasing daily, with high rates.^(1)^

It is known that multiple pregnancies require special prenatal care due to the high maternal-fetal risk involved. The risk of COVID-19 infection in twin pregnancy is similar to that in singleton pregnancy. Chorionicity is determined in the first trimester and classifies twin pregnancies into two groups. According to the American College of Obstetricians and Gynecologists (ACOG) and the International Society of Ultrasound in Obstetrics & Gynecology (ISUOG), as from 16 weeks of gestation, monochorionic pregnancies should be monitored by ultrasound every 2 weeks, while dichorionic pregnancies should be monitored every 4 weeks.^(2)^

Twin-to-twin transfusion syndrome occurs in 10% to 15% of monochorionic pregnancies, requires early diagnosis, and has a mortality rate of 90% and morbidity of approximately 50% in the surviving fetus. The risk of fetal death is six-fold and eight-fold higher in monochorionic and dichorionic pregnancies, respectively, after 24 weeks of gestation, when compared to singleton pregnancies. The frequency of pre-eclampsia is two to three-fold higher in twin pregnancies. For this and other risks, many obstetric facilities define the intervals between visits based on ultrasound images.^(3)^ However, obstetric care had to be reorganized due to COVID-19 pandemic. Physical contact must be avoided, and pregnant women and medical staff must wear personal protective equipment. The ACOG suggested prenatal care for singleton pregnancies should take place at four main moments. The first at approximately 12 weeks, when the first trimester scan is performed, and the second at around 20 gestational weeks, for the second trimester scan. The third moment takes place at 24 to 28 weeks, when it is important to screen for gestational diabetes, and, finally, at approximately 34 weeks, to assess vitality in the third trimester.^(4)^

In twin pregnancies, the performance of ultrasounds is even more special, since this condition presents a higher frequency of aneuploidies. The period between 24 and 28 weeks also applies to multiple pregnancies because, in addition to the higher frequency of gestational diabetes in this group, some types of twin pregnancies also have a higher prevalence of heart defects. Therefore, ISUOG recommends performing fetal echocardiography in all monochorionic pregnancies. Fetal vitality in the third trimester deserves more attention, especially in multiple pregnancies, since this group has a higher incidence of selective intrauterine growth restriction and weight discordance among fetuses.^(2,3)^Despite the recommendation of presential assessment by ACOG, the usual frequency of obstetric visits could be maintained by means of the recent development of telehealth. To reduce exposure of patients and the transmission of COVID-19, virtual consultations allow maintaining quality and safety of prenatal care, as well as detection of adverse outcomes, despite the distance.^(5)^ In the case of multiple pregnancies, the patient’s weight control and blood pressure measurements can be performed at home and informed to the physician through telehealth. The prenatal care physician can talk directly to the patient and send reports, prescriptions and exam requests with a digital signature, and follow up progression of pregnancy.^(6,7)^

According to the ACOG algorithm, women with a history of contact with COVID-19 patients should maintain routine prenatal care. In case of symptomatic patients, the ACOG divided them into three risk groups (low, moderate and high). For those in the low risk group, presenting mild symptoms and no comorbidities, self-isolation is recommended. The moderate risk group, with comorbidities, obstetric conditions or unable to perform selfcare, should receive care at outpatient’s setting. Those at high risk, with severe symptoms, such as respiratory distress, fever, radiological evidence of pneumonia, should be promptly referred to an emergency department.^(8)^

The use of corticosteroids in multiple pregnancies between 24 and 34 weeks, for lung maturation and reduction of other sequelae of prematurity, is well accepted in all obstetric guidelines, for stable pregnancy. The incidence of premature birth has increased in pregnant women with COVID-19. The royal college of obstetricians and gynaecologists (RCOG) recommends an ultrasound should be performed 14 days after the end of acute illness, whereas the ACOG recommends a third-trimester ultrasound for pregnant women infected in the previous two gestational trimesters.^(8-10)^

Although twin pregnancies have an earlier termination in relation to singleton (approximately 38 weeks for dichorionic, 36 weeks for monochorionic, and 32 to 34 weeks for monoamniotic), it is recommended that delivery should not be abbreviated during the COVID-19 infection, if there is no justified maternal-fetal morbidity. Waiting for a negative result for coronavirus test can be an important decision for better maternal-fetal and neonatal care during delivery and breastfeeding assistance.^(11)^
