# Supplementary material for: Multiple pregnancy in SARS-CoV-2 outbreak: the prenatal care challenge
Source: Einstein (Sao Paulo). 2020 Sep 16;18:eCE5990. doi: 10.31744/einstein_journal/2020CE5990 (PMC9586423; doi:10.31744/einstein_journal/2020CE5990)
Supplement: Translation

# Translation: Gestação múltipla no surto de SARS-CoV-2: o desafio do pré-natal

**Authors:** Eduardo Felix Martins Santana, Julio Elito

**Affiliations:** 1 Brasil Faculdade Israelita de Ciências da Saúde Albert Einstein, Hospital Israelita Albert Einstein, São Paulo, SP, Brasil.; 2 Brasil Hospital Israelita Albert Einstein, São Paulo, SP, Brasil.; 3 Brasil Escola Paulista de Medicina, Universidade Federal de São Paulo, São Paulo, SP, Brasil.

Caro Editor,

A história da humanidade mudou em dezembro de 2019, quando um novo vírus foi detectado na cidade de Wuhan, na China. Milhões de indivíduos foram rapidamente infectados no mundo todo, e o novo coronavírus, inicialmente chamado doença pelo coronavírus 2019 (COVID-19), trouxe um impacto enorme ao causar a síndrome respiratória aguda grave; por isso passou a ser denominado coronavírus da síndrome respiratória aguda grave 2 (SARS-CoV-2). Com transmissão rápida, em março de 2020, a Organização Mundial da Saúde (OMS) declarou a infecção como uma pandemia. Indivíduos em todos os continentes foram afetados, e o número de mortes tem aumentado diariamente, com altas taxas.^(1)^

Sabe-se que gestações múltiplas requerem atendimento pré-natal especial, devido ao maior risco materno-fetal. O risco da infecção pela COVID-19 em gestação gemelar é semelhante ao da gestação única. A placentação é determinada no primeiro trimestre e classifica as gestações gemelares em dois grupos. De acordo com o *American College of Obstetricians and Gynecologists* (ACOG) e a *International Society of Ultrasound in Obstetrics & Gynecology* (ISUOG), a partir de 16 semanas, as gestações monocoriônicas devem ser monitoradas por ultrassom, a cada 2 semanas, ao passo que as dicoriônicas, a cada 4 semanas.^(2)^

A síndrome de transfusão feto-fetal ocorre em 10% a 15% das gestações monocoriônicas, requer diagnóstico precoce e tem taxa de mortalidade de 90% e morbidade de aproximadamente 50% no feto sobrevivente. O risco de morte fetal é seis e oito vezes maior nas gestações monocoriônicas e dicoriônicas, respectivamente, após 24 semanas de gestação, quando comparado ao das gestações únicas. A frequência de pré-eclâmpsia é duas a três vezes maior nas gestações gemelares. Para esse e outros riscos, muitos centros obstétricos definem os intervalos entre as consultas com base nas imagens ultrassonográficas.^(3)^No entanto, os cuidados obstétricos precisaram ser reorganizados, devido à pandemia de COVID-19. Deve-se evitar o contato físico, e as gestantes e a equipe médica devem usar equipamentos de proteção individual. O ACOG sugeriu que a atenção pré-natal para gestações únicas ocorra em quatro momentos principais. O primeiro, com aproximadamente 12 semanas, quando se realiza o ultrassom do primeiro trimestre, e o segundo, com cerca de 20 semanas, para o ultrassom do segundo trimestre. O terceiro momento acontece com 24 a 28 semanas, quando é importante rastrear diabetes gestacional e, finalmente, com cerca de 34 semanas, para avaliar vitalidade no terceiro trimestre.^(4)^

Nas gestações gemelares, a realização de ultrassonografias é ainda mais especial, pois esta situação presenta maior frequência de aneuploidias. O período entre 24 e 28 semanas também se aplica a gestações múltiplas, pois, além da maior frequência de diabetes gestacional neste grupo, alguns tipos de gestações gemelares também têm maior prevalência de defeitos cardíacos. Portanto, a ISUOG recomenda realizar ecocardiografia fetal em todas as gestações monocoriônicas. A vitalidade fetal no terceiro trimestre merece mais atenção, especialmente nas gestações múltiplas, já que esse grupo tem maior incidência de restrição de crescimento intrauterino e discrepância ponderal entre os fetos.^(2,3)^Apesar da recomendação do ACOG para avaliação presencial, a frequência usual de consultas obstétricas deve ser mantida, por meio do desenvolvimento recente da telessaúde. Para reduzir a exposição das pacientes e a transmissão da COVID-19, as consultas virtuais permitem manter a qualidade e a segurança do cuidado pré-natal e detectar os desfechos adversos, apesar da distância.^(5)^ No caso de gestações múltiplas, o controle de peso e as mensurações de pressão arterial da paciente devem ser realizados em casa e informados ao médico por meio da telessaúde. O médico do atendimento pré-natal pode conversar diretamente com a paciente e enviar relatórios, prescrições e solicitações de exames com assinatura digital, acompanhando a evolução da gravidez.^(6,7)^

Conforme o algoritmo do ACOG, as mulheres com história de contato com pacientes com COVID-19 devem manter o atendimento pré-natal de rotina. No caso de pacientes sintomáticas, o ACOG dividiu em três grupos de risco (baixo, moderado e alto). Para aquelas no grupo de baixo risco, com sintomas leves e sem comorbidades, recomenda-se isolamento. O grupo de risco moderado, com comorbidades, doenças obstétricas ou incapaz de realizar autocuidado, deve ser atendido no nível ambulatorial. Aquelas com alto risco, com sintomas graves, como desconforto respiratório, febre e evidências radiológicas de pneumonia, devem ser encaminhadas imediatamente ao pronto-socorro.^(8)^

O uso de corticoides nas gestações múltiplas, entre 24 e 34 semanas, para maturação pulmonar e redução de outras sequelas da prematuridade, é bem aceito em todas as diretrizes obstétricas, nas gestações estáveis. A incidência de partos prematuros aumentou nas gestantes com COVID-19. O *Royal College of Obstetricians and Gynaecologists* (RCOG) recomenda realizar uma ultrassonografia 14 dias após o final da doença aguda, enquanto o ACOG recomenda um ultrassom do terceiro trimestre para as gestantes infectadas nos dois trimestres anteriores.^(8-10)^

Apesar das gestações gemelares terem um final mais precoce em relação às gestações únicas (aproximadamente 38 semanas para dicoriônicas, 36 semanas para monocoriônicas e 32 a 34 semanas para as monoamnióticas), recomenda-se que o parto não seja antecipado durante a infecção por COVID-19, caso não haja morbidade materno-fetal que o justifique. Esperar por um resultado negativo no teste para coronavírus pode ser uma decisão importante para melhores cuidados materno-fetais e neonatais, durante o parto e o aleitamento materno.^(11)^
